# Exercise self-management behavioral phenotypes in type 2 diabetes: a multicenter cross-sectional study

**DOI:** 10.3389/fpubh.2026.1892333

**Published:** 2026-09-22

**Authors:** Xi Chen, Liya Zhang, Huili Jiang, Yuhao Chen, Fangping Xie, Xiaowen Zhai, Yifan Liu, Chunyan Zhao, Fei He, Qian Zhou, Di Zhang, Bin Xue, Fanglei Xu, Suwen Feng

**Affiliations:** 1 Department of Clinical Research Unit, Shanghai Tongji Hospital, Tongji University School of Medicine, Shanghai, China; 2 Department of Nursing, Shanghai Tongji Hospital, Tongji University School of Medicine, Shanghai, China; 3 Department of Cardiology, Shanghai Tongji Hospital, Tongji University School of Medicine, Shanghai, China; 4 Department of Clinical Research Ward, Cancer Center, Shanghai Tongji Hospital, Tongji University School of Medicine, Shanghai, China; 5 Department of Hematology, Shanghai Tongji Hospital, Tongji University School of Medicine, Shanghai, China; 6 Department of Nursing, Shanghai East Hospital, Tongji University School of Medicine, Shanghai, China

**Keywords:** complications, exercise management, knowledge-attitude-practice (KAP), latent profile analysis, type 2 diabetes

## Abstract

**Background:**

Exercise management is important in type 2 diabetes mellitus (T2DM). However, individual variability in exercise-related knowledge, attitudes, and practices (KAP) is significantly associated with behavioral adherence yet is often overlooked in average-based analyses. This study applied Latent profile analysis (LPA) to characterize this heterogeneity and explored associations between the resulting behavioral profiles and clinical indicators.

**Methods:**

We conducted a multicenter cross-sectional study of 516 adults with T2DM in China. LPA was performed using standardized exercise-related KAP indicators. Candidate solutions were evaluated using fit indices, classification diagnostics, interpretability, and bootstrap reproducibility. Clinical associations were examined using adjusted regression and posterior-probability pseudo-class analyses.

**Results:**

Four exploratory profiles were identified: Favorable Engagement (7.0%), Average Engagement (59.7%), Knowledge-Constrained (21.7%), and Attitude–Practice Constrained (11.6%). After covariate adjustment, BMI differed across profiles; only the Average Engagement contrast remained significant after accounting for classification uncertainty. Glycemic indicators and diabetes-related complications did not differ robustly. The adjusted association with other chronic disease was sensitive to classification uncertainty.

**Conclusion:**

Exercise self-management heterogeneity included distinct knowledge-related and attitude–practice constraints. These exploratory patterns may inform tailored support, but profile instability and cross-sectional data require independent prospective validation.

## 1 Introduction

Type 2 diabetes mellitus (T2DM) (1) has become an increasingly severe global public health challenge, with its prevalence rising sharply over the past few decades. According to data from the International Diabetes Federation (IDF), the number of people with diabetes worldwide surged from 153 million in 1980 to 537 million in 2021, and it is expected to reach approximately 643 million by 2030 (2). China faces the world’s highest diabetes burden, with over 118 million affected individuals accounting for approximately 22% of global cases (3). This high prevalence imposes a substantial clinical and economic burden. The situation is worsened by high rates of diabetes-related complications, including cardiovascular disease, chronic kidney disease, retinopathy, and neuropathy, which contribute to rising healthcare costs and amplify health disparities (4, 5).

Lifestyle interventions (6), particularly physical activity and exercise management, play a critical role in diabetes control. In adults with impaired glucose tolerance who had not yet developed diabetes, the original US Diabetes Prevention Program and Finnish Diabetes Prevention Study showed that intensive lifestyle intervention reduced the incidence of T2DM by approximately 58% (7, 8). Nonetheless, translating physical activity recommendations into sustained behavior change remains challenging. Variability in exercise adherence is shaped by a range of factors, including health knowledge, risk perception, cultural beliefs, socioeconomic conditions, and access to educational resources (9). Such behavioral variation may be associated with differences in glycemic control and complication burden (10, 11).

The Knowledge–Attitude–Practice (KAP) framework, which posits that knowledge shapes beliefs, attitudes, and ultimately behavior, is widely used to study self-management in chronic diseases like diabetes (12–17). Most previous studies have used variable-centered methods, which may obscure meaningful between-person heterogeneity. As a result, the findings offer limited guidance for creating personalized interventions (18). Evidence linking exercise-related KAP patterns with metabolic indicators, comorbidity burden, and diabetes-related complications remains limited (19, 20).

To address these limitations, researchers are increasingly turning to person-centered analytical strategies, such as Latent profile analysis (LPA) (21–24). This method identifies distinct behavioral subgroups based on multivariate response patterns, moving beyond group averages to reveal clinically significant phenotypes masked in broader analyses. Furthermore, LPA has been applied in diverse health and behavioral settings (25–29).

This study employed LPA within the KAP framework to characterize exercise management profiles among patients with T2DM in a Chinese multicenter cohort. We compared two- to four-profile solutions, assessed their stability and classification uncertainty, and examined adjusted associations with clinical outcomes. We hypothesized that more detailed profiles might reveal distinct behavioral patterns.

## 2 Methods

### 2.1 Study design and participants

This cross-sectional study was conducted between September and December 2022 in Shanghai and Hainan province, China. A total of 516 adults diagnosed with T2DM were consecutively enrolled from four tertiary hospitals (Shanghai Tongji Hospital, Tongji University School of Medicine; Shanghai East Hospital, Tongji University School of Medicine; Ren Ji Hospital, Shanghai Jiao Tong University School of Medicine and Dongfang People’s Hospital in Hainan Province) and four community health service centers in Shanghai (Nicheng Health Service Center, Fangsong Health Service Center, Jiuting Health Service Center, and Chedun Health Service Center). Ethical approval was granted by the Ethics Committee of Shanghai East Hospital, Tongji University School of Medicine (2020–026). Written informed consent was obtained from all participants prior to data collection (30). The study was conducted in accordance with the principles of the Declaration of Helsinki.

Inclusion criteria (31) were: (1) diagnosis of T2DM according to the 2020 Chinese Guidelines for Diabetes Diagnosis and Treatment; (2) age ≥ 18 years; (3) disease duration ≥ 6 months; and (4) normal mobility without exercise-limiting disabilities.

Exclusion criteria included: (1) acute diabetic complications or severe systemic comorbidities; (2) pregnancy or lactation; and (3) cognitive impairment or major psychiatric disorders.

### 2.2 Instruments and variable measurement

The survey instrument was developed based on the theoretical framework of the Health Belief Model (HBM), originally proposed by Rosenstock and later expanded by Becker and Maiman (32, 33). The HBM conceptualizes health behavior as a function of perceived susceptibility, perceived severity, perceived benefits, perceived barriers, cues to action, and self-efficacy.

The questionnaire used in this study was derived from a validated Chinese instrument developed by Feng et al. (30), which was specifically designed to assess exercise-related knowledge, attitudes, and practices among patients with type 2 diabetes mellitus. In the present study, the structure and item content were retained, with minor linguistic refinement to ensure applicability to the current study population.

#### 2.2.1 General information questionnaire

A structured, investigator-developed questionnaire was used to collect demographic characteristics, lifestyle behaviors, and clinical history. Information included age, sex, education level, occupation, working status, marital status, medical insurance coverage, residential status, monthly household income, diabetes duration, hypoglycemia history, presence of chronic diseases and diabetes-related complications, treatment modalities, and access to exercise-related support services. Detailed item content is provided in the Supplementary material 1.

#### 2.2.2 Exercise belief questionnaire

Exercise-related beliefs were assessed using a 21-item questionnaire grounded in the Health Belief Model, encompassing six theoretical domains: perceived susceptibility, perceived severity, perceived benefits, perceived barriers, cues to action, and self-efficacy. All items were rated on a five-point Likert scale.

The internal consistency of the scale in the present sample was acceptable to excellent across subscales (Cronbach’s α = 0.744–0.923), consistent with the reliability reported in the original validation study.

#### 2.2.3 Exercise self-management behavior scale

Exercise behaviors over the preceding 6 months were measured using an 18-item, three-domain scale covering: (1) planning and monitoring, (2) exercise execution, and (3) safety management. The scale was originally developed and validated by Feng et al. (2024) and demonstrated high internal consistency (Cronbach’s α = 0.887) and satisfactory content validity (I-CVI = 0.83–1.00; S-CVI = 0.94) in the original study.

In the present study, the scale was applied without modification. Higher scores reflected better adherence to exercise self-management behaviors.

### 2.3 Construction of KAP variables

Following standard operationalization of the Knowledge-Attitude-Practice framework (34):

Knowledge: The five-level awareness item was originally coded from 1 (very well understood) to 5 (completely unknown) and was reverse-coded so that higher values indicated greater awareness. Receipt of diabetes exercise health education was coded 1 (no) and 2 (yes), so higher values indicated educational exposure. Each component was then standardized separately and the two *z* scores were summed with equal weight. Receipt of education reflects exposure or access rather than direct factual knowledge. In a sensitivity analysis, Knowledge was represented only by the reverse-coded, standardized awareness item. Full wording and coding are provided in Supplementary material 1.

Attitude: Scores were estimated from eight Health Belief Model items measuring perceived benefits (3 items), perceived barriers (2 reverse-scored items), and self-efficacy (3 items). A confirmatory factor analysis was fitted using robust maximum likelihood estimation. Regression factor scores were calculated for the three domains and summed to obtain the Attitude indicator. The CFA showed mixed model fit, with scaled χ^2^(17) = 65.22, *p* < 0.001, robust CFI = 0.977, robust TLI = 0.962, robust RMSEA = 0.078 (95% CI 0.059–0.099), and SRMR = 0.029. Standardized factor loadings ranged from 0.628 to 1.143, and one loading exceeded 1, indicating an inadmissible estimate and potential model misspecification.

Practice: It was represented by total scores from the exercise self-management behavior scale (Cronbach’s α = 0.89).

These three domains were selected because they most directly represented participants’ evaluations of and confidence in exercise engagement. Perceived susceptibility, perceived severity, and cues to action were not included in the Attitude indicator, resulting in a narrower operational definition of Attitude.

All KAP constructs were normalized prior to modeling.

### 2.4 Clinical outcomes and covariates

Clinical outcomes included four continuous variables—BMI, waist-to-hip ratio (WHR), fasting blood glucose (FBG), and HbA1c—and two binary variables: the presence of any recorded diabetes-related complications and the presence of any other chronic disease. Individual complication subtypes were analyzed separately as exploratory outcomes because of sparse event counts and multiple comparisons.

All adjusted outcome models included age, sex, diabetes-duration category, education level, household income, use of oral glucose-lowering medication, and insulin therapy. Models for any diabetes-related complication and any other chronic disease additionally included BMI. A participant-level recruitment-center identifier was unavailable in the analytic dataset; therefore, center fixed effects, multilevel models, and cluster-robust standard errors could not be estimated.

Other sociodemographic, behavioral, treatment, and exercise-support variables were summarized descriptively across profiles and were not interpreted as consequences of profile membership.

### 2.5 Statistical analysis

#### 2.5.1 Data preprocessing

Data coding and missingness were checked before analysis. One missing Attitude-item value was handled using full-information maximum likelihood in the CFA, whereas clinical models used available observations for each outcome. KAP indicators were standardized as z scores before LPA.

#### 2.5.2 Confirmatory factor analysis

Model fit was assessed using χ^2^/df, comparative fit index (CFI), Tucker-Lewis index (TLI), root mean square error of approximation (RMSEA), and standardized root mean square residual (SRMR), and the three regression factor scores were summed to construct the Attitude indicator. Standardized loadings were examined for inadmissible estimates.

#### 2.5.3 Latent profile analysis

LPA was conducted using the tidyLPA package with the standardized Knowledge-related, Attitude, and Practice indicators. The primary analysis compared two-, three-, and four-profile solutions under an equal-variance, zero-covariance parameterization (Model 1). Model selection considered AIC, BIC, SABIC, BLRT, entropy, class size, average posterior probabilities, interpretability, and bootstrap reproducibility. To examine sensitivity to model parameterization, two- to four-profile solutions were additionally fitted under an unequal-variance, zero-covariance parameterization (Model 2) and an equal-variance, equal-covariance parameterization (Model 3). These alternative parameterizations were treated as supplementary model-specification checks and were not used to redefine the primary profile solution. Because the selection criteria did not consistently favor one solution, the retained four-profile Model 1 solution was interpreted as exploratory.

#### 2.5.4 Adjusted associations with clinical outcomes

Unadjusted characteristics were summarized as mean ± SD or n (%). Profile associations with BMI, WHR, FBG, and HbA1c were examined using multivariable linear regression, whereas diabetes-related complications and other chronic disease were examined using logistic regression. Models adjusted for age, sex, diabetes duration, education, household income, oral glucose-lowering medication, and insulin therapy; binary disease models additionally adjusted for BMI. Favorable Engagement was the reference profile.

Overall profile effects were evaluated using nested-model *F* tests for continuous outcomes and likelihood-ratio tests for binary outcomes. Profile contrasts were reported as β coefficients or ORs with 95% confidence intervals. Complication-subtype analyses used full profile denominators, Wilson 95% confidence intervals, Fisher’s exact tests, and Benjamini–Hochberg correction.

#### 2.5.5 Analysis accounting for classification uncertainty

Given the moderate entropy of the four-profile solution, classification uncertainty was addressed using posterior-probability pseudo-class analysis. Profile membership was sampled from each participant’s posterior probabilities to generate 100 pseudo-class datasets. The adjusted regression models were fitted in each dataset, and estimates were pooled using Rubin’s rules. Results were reported as β coefficients or ORs with 95% confidence intervals. This analysis assessed the sensitivity of clinical associations to classification uncertainty and was not used for profile enumeration.

#### 2.5.6 Bootstrap reproducibility and knowledge-specification sensitivity analyses

The four-profile model was evaluated using 500 participant-level bootstrap samples. Complete solutions were retained, and label switching was addressed by matching bootstrap profiles to the original solution based on Euclidean distance across the three standardized KAP indicators. Profile proportions, entropy, and profile reproducibility were summarized across bootstrap samples.

Sensitivity analyses were conducted by removing the exercise-education indicator and defining the knowledge-related indicator using only the five-level awareness item. Two- to four-profile solutions were then re-estimated using the same model specifications and selection criteria.

A fitted solution was considered complete when estimation returned a finite log-likelihood and all specified profiles contained at least one participant under maximum-posterior-probability assignment.

### 2.6 Software

All analyses were performed using R software (version 4.4.2; R Foundation for Statistical Computing, Vienna, Austria). The principal packages included tidyLPA (version 1.1.0), lavaan (version 0.6–20), dplyr (version 1.1.4), and ggplot2 (version 4.0.1). A root seed of 20,260,805 was used for the pseudo-class and bootstrap analyses.

## 3 Results

### 3.1 Baseline characteristics of participants

Table 1 summarizes the baseline characteristics of the 516 participants. The mean age was 64.4 ± 12.0 years, and 283 (54.8%) participants were female. The mean BMI was 24.7 ± 4.2 kg/m^2^, mean waist circumference was 84.4 ± 16.5 cm, and mean WHR was 0.91 ± 0.16.

**Table 1:** Baseline characteristics of participants (*N* = 516).

Characteristic	Value
Demographics	
Age, years, mean ± SD	64.4 ± 12.0
Female sex, *n* (%)	283 (54.8)
Anthropometric measures	
BMI, kg/m^2^, mean ± SD	24.7 ± 4.2
Waist circumference, cm, mean ± SD	84.4 ± 16.5
Waist-to-hip ratio, mean ± SD	0.91 ± 0.16
Socioeconomic characteristics	
Junior high school education or above, n (%)	377 (73.0)
Retired, *n* (%)	374 (72.3)
Clinical history	
Family history of diabetes, *n* (%)	230 (44.6)
Any other chronic disease, *n* (%)	268 (52.0)
Hypertension	230 (44.6)
Coronary artery disease	71 (13.8)
Glycemic indicators	
Fasting blood glucose, mmol/L, mean ± SD	7.36 ± 1.96
HbA1c, %, mean ± SD	7.21 ± 1.76
Diabetes-related complications	
Any complication, *n* (%)	130 (25.2)
Retinopathy	38 (7.4)
Peripheral neuropathy	49 (9.5)
Nephropathy	40 (7.8)
Treatment behaviors	
Dietary control, *n* (%)	391 (75.8)
Oral glucose-lowering medications, *n* (%)	381 (73.8)
Exercise, *n* (%)	298 (57.8)
Insulin therapy, *n* (%)	312 (60.5)

Overall, 377 participants (73.0%) had completed at least junior high school education, and 374 (72.3%) were retired. A family history of diabetes was reported by 230 (44.6%) participants. Other chronic diseases were reported by 268 (52.0%), with hypertension (230, 44.6%) and coronary artery disease (71, 13.8%) being the most frequently recorded conditions.

Mean FBG was 7.36 ± 1.96 mmol/L, and mean HbA1c was 7.21 ± 1.76%. Diabetes-related complications were recorded in 130 participants (25.2%). The most frequent subtypes were peripheral neuropathy (49, 9.5%), nephropathy (40, 7.8%), and retinopathy (38, 7.4%).

Oral glucose-lowering medication was used by 381 participants (73.8%), insulin therapy by 312 (60.5%), and both treatments by 212 (41.1%). Dietary control was reported by 391 participants (75.8%), and 298 (57.8%) reported regular physical activity.

### 3.2 Identification of latent exercise management profiles

In the primary Model 1 analysis, statistical evidence favored three or four profiles over two profiles but did not identify a single unequivocal solution (Table 2). For the two-, three-, and four-profile solutions, BIC values were 4359.23, 4172.74, and 4186.01; entropy values were 0.824, 0.852, and 0.714; and BLRT *p* values were 0.0099 for all three solutions. The four-profile solution had the lowest AIC (4109.58), whereas the three-profile solution had the lowest BIC and SABIC (4172.74 and 4128.30, respectively) and higher entropy. Alternative variance–covariance parameterizations also favored three- or four-profile solutions over the corresponding two-profile solutions but did not provide unequivocal support for the selected four-profile Model 1 solution. Complete model-specification results are presented in Supplementary Table 1.

**Table 2:** Fit indices for latent profile analysis models.

**Model**	**Classes**	**AIC**	**BIC**	**SABIC**	**Entropy**	**LogLik**	**BLRT p**
1	2	4316.77	4359.23	4327.49	0.824	−2148.39	0.0099
1	3	4113.30	4172.74	4128.30	0.852	−2042.65	0.0099
**1**	**4**	**4109.58**	**4186.01**	**4128.88**	**0.714**	**−2036.79**	**0.0099**

Model 1 assumes equal within-profile variances and zero within-profile covariances. BLRT compares the k-profile solution with the k − 1-profile solution. The selected four-profile solution is followed by bold. AIC, Akaike information criterion; BIC, Bayesian information criterion; SABIC, sample-size-adjusted Bayesian information criterion; BLRT, bootstrap likelihood-ratio test.

The four-profile solution had moderate entropy (0.714). Average posterior probabilities were 0.848, 0.823, 0.965, and 0.658 for Profiles 1–4, respectively. Classification was clearest for the Knowledge-Constrained Profile and weakest for the Attitude–Practice Constrained Profile.

The four-profile solution was retained as an exploratory representation because it separated a low-knowledge pattern from a combined attitude and practice constraint that were merged in simpler solutions. It was not interpreted as statistically or clinically definitive.

Four profiles were identified (Figure 1): Favorable Engagement (*n* = 36, 7.0%), Average Engagement (*n* = 308, 59.7%), Knowledge-Constrained (*n* = 112, 21.7%), and Attitude–Practice Constrained (*n* = 60, 11.6%). The Knowledge-Constrained Profile had the lowest Knowledge-related score with near-average Attitude and mildly below-average Practice. The Attitude–Practice Constrained Profile had the lowest Attitude and Practice scores.

**Figure 1:**
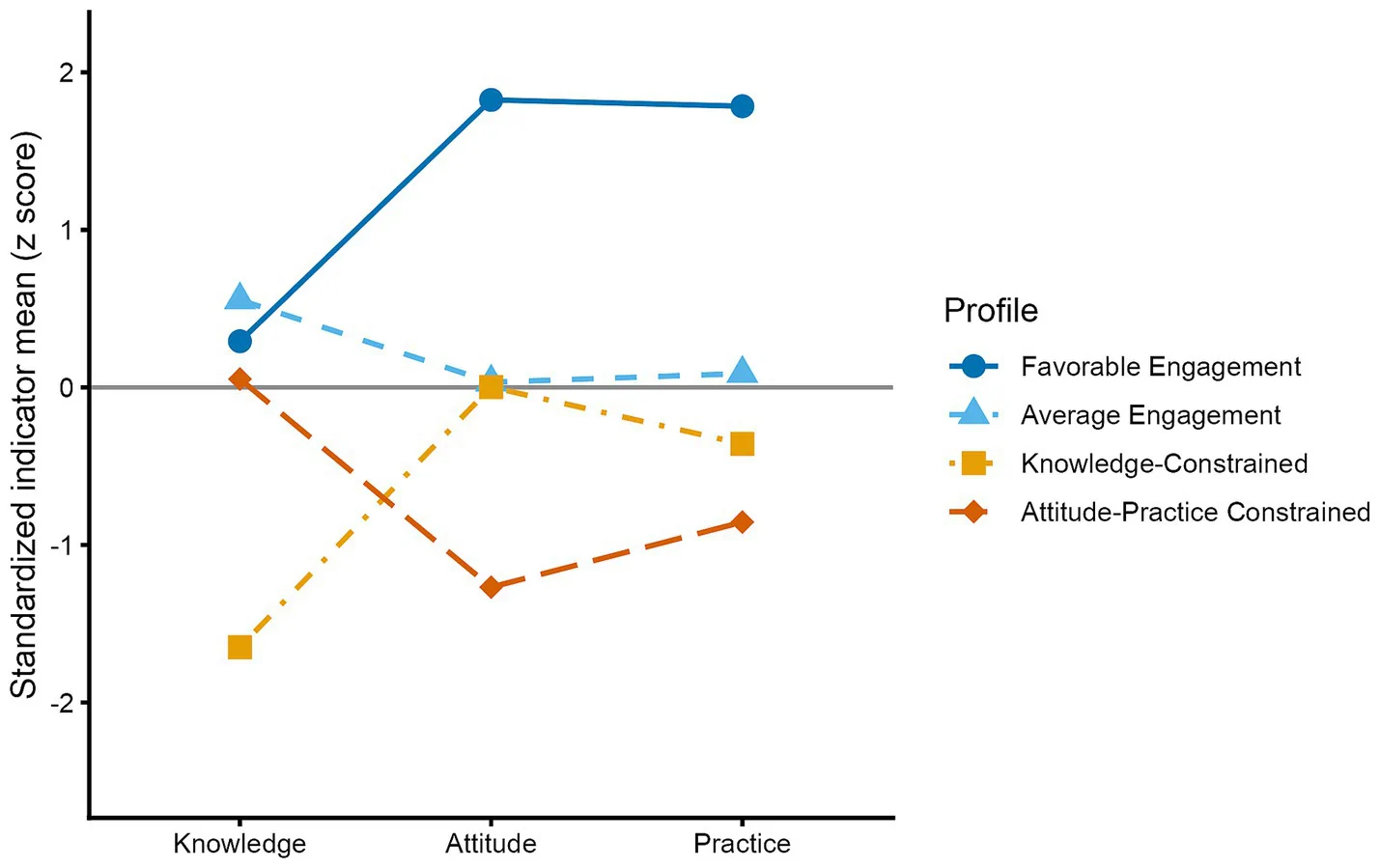
Exercise self-management profiles. Standardized means of the knowledge-related, attitude, and practice indicators across the four exploratory profiles. Positive and negative values indicate scores above and below the sample mean, respectively. Profile labels describe relative behavioral patterns rather than an ordered severity scale.

### 3.3 Unadjusted clinical characteristics across profiles

Unadjusted clinical outcomes across assigned profiles are presented in Figures 2, 3. Mean BMI was 22.34 ± 2.91, 25.03 ± 4.14, 24.08 ± 3.99, and 25.15 ± 5.03 kg/m^2^ across Profiles 1–4, respectively. Descriptive values for WHR, FBG, and HbA1c are provided in Supplementary Table 2.

**Figure 2:**
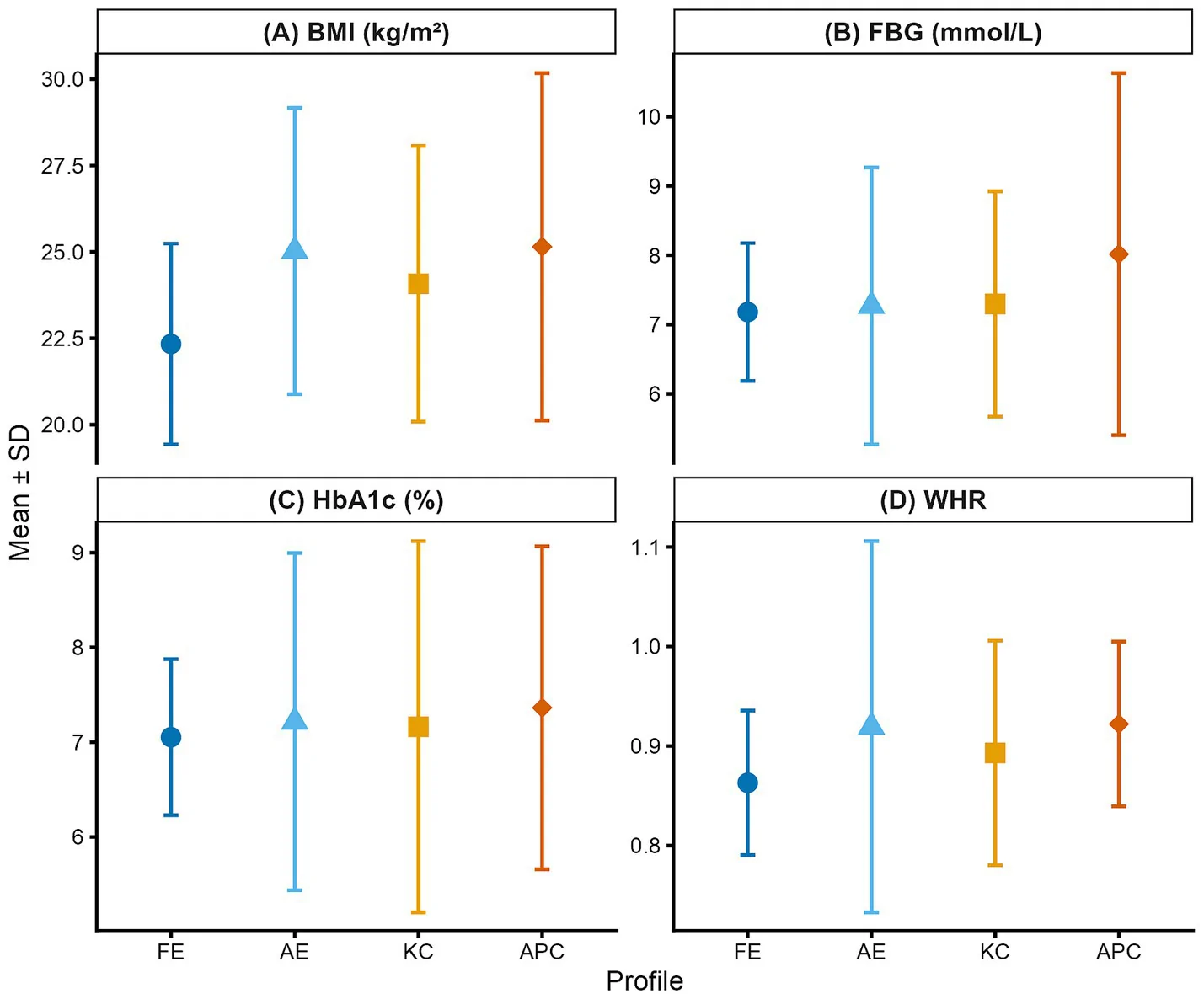
Clinical measures across profiles. Unadjusted means and standard deviations of clinical measures across the assigned profiles: **(A)** body mass index, **(B)** waist-to-hip ratio, **(C)** fasting blood glucose, and **(D)** HbA1c. Error bars represent standard deviations. FE, Favorable Engagement; AE, Average Engagement; KC, Knowledge-Constrained; APC, Attitude–Practice Constrained.

**Figure 3:**
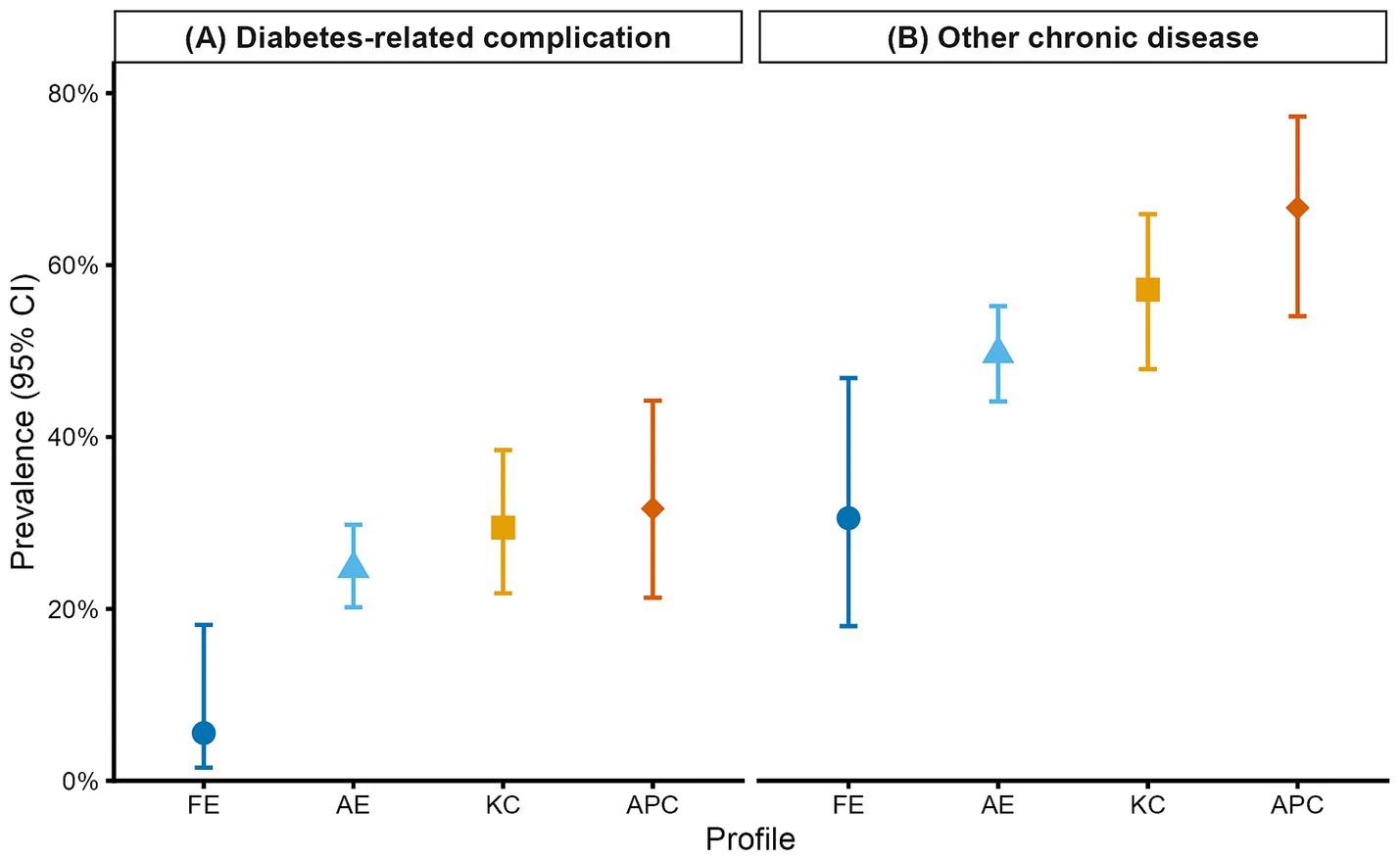
Disease prevalence across profiles. Unadjusted prevalence and Wilson 95% confidence intervals for **(A)** any recorded diabetes-related complications and **(B)** any other chronic disease across the assigned profiles. FE, Favorable Engagement; AE, Average Engagement; KC, Knowledge-Constrained; APC, Attitude–Practice Constrained.

The prevalence of diabetes-related complications was 5.6% (2/36), 24.7% (76/308), 29.5% (33/112), and 31.7% (19/60) across Profiles 1–4. The corresponding prevalence of other chronic disease was 30.6% (11/36), 49.7% (153/308), 57.1% (64/112), and 66.7% (40/60). Wilson 95% confidence intervals are shown in Figure 3 and Supplementary Table 3.

Clinical and demographic characteristics were treated as descriptive covariates rather than consequences of profile membership.

### 3.4 Adjusted associations with clinical indicators

After adjustment, the overall association between profile membership and BMI was significant (*p* = 0.002). Relative to Favorable Engagement, BMI was higher in Average Engagement (*β* = 2.70, 95% CI 1.18–4.23), Knowledge-Constrained (*β* = 1.79, 95% CI 0.12–3.45), and Attitude–Practice Constrained (*β* = 2.81, 95% CI 1.00–4.63). Overall profile effects were not significant for WHR (*p* = 0.108), FBG (*p* = 0.073), or HbA1c (*p* = 0.611). The adjusted overall profile effect was not significant for diabetes-related complications (*p* = 0.437). The adjusted overall test for any other chronic disease was significant in the maximum-posterior-probability assignment analysis (*p* = 0.040). Relative to Favorable Engagement, the Attitude–Practice Constrained Profile had an OR of 3.62 (95% CI 1.39–9.47). However, this association did not persist in the posterior-probability pseudo-class analysis accounting for classification uncertainty.

Profile-specific estimates for diabetes-related complications were imprecise, and all 95% confidence intervals included the null. Relative to Favorable Engagement, the adjusted ORs were 2.15 (95% CI 0.46–10.01) for Average Engagement, 2.82 (95% CI 0.58–13.64) for Knowledge-Constrained, and 2.81 (95% CI 0.55–14.40) for the Attitude–Practice Constrained Profile.

In 100 posterior-probability pseudo-class datasets, BMI remained higher only in Average Engagement (*β* = 1.96, 95% CI 0.25–3.67) than in Favorable Engagement. No uncertainty-aware contrast was significant for WHR, FBG, HbA1c, diabetes-related complications, or other chronic disease. Complete adjusted results are provided in Supplementary Tables 4, 5.

Complication-subtype events were sparse (Figure 4), and no subtype comparison was significant after false-discovery-rate correction. Detailed results are provided in Supplementary Table 6.

**Figure 4:**
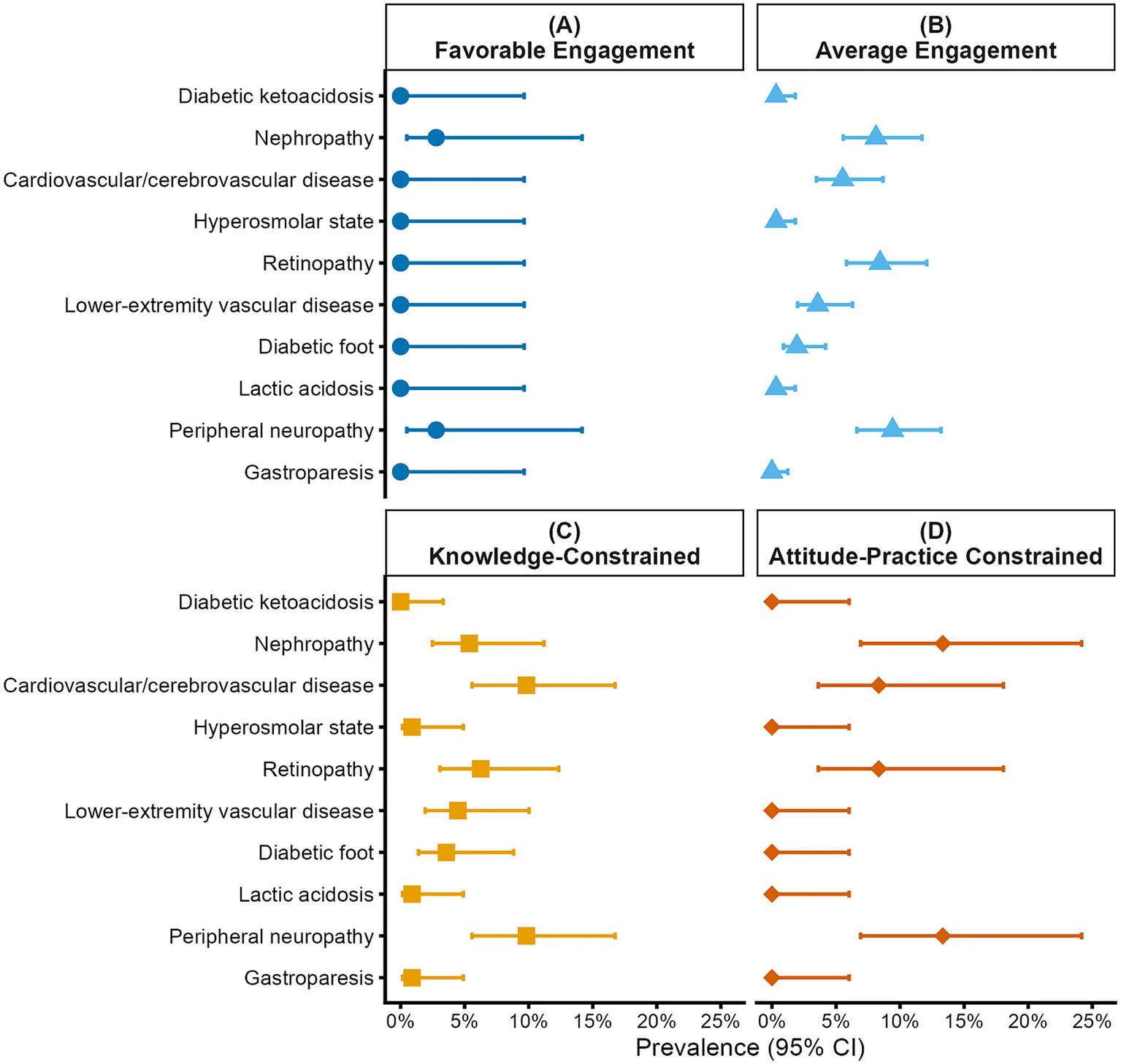
Complication subtypes across profiles. Prevalence and Wilson 95% confidence intervals for recorded diabetes-related complication subtypes in the **(A)** Favorable Engagement, **(B)** Average Engagement, **(C)** Knowledge-Constrained, and **(D)** Attitude–Practice Constrained Profiles. Subtype analyses were exploratory because event counts were sparse, and no difference remained significant after false-discovery-rate correction. DKA, diabetic ketoacidosis.

### 3.5 Bootstrap reproducibility and knowledge-specification sensitivity

Of 500 bootstrap resamples, 495 yielded complete four-profile solutions with at least one participant assigned to each profile. After label alignment, the mean profile proportions were 6.1, 41.4, 21.1, and 31.4%, compared with empirical proportions of 7.0, 59.7, 21.7, and 11.6%, respectively. The corresponding 95% percentile intervals were 1.4–10.8%, 9.4–64.2%, 16.9–25.2%, and 6.9–67.4% (Figure 5). Mean bootstrap entropy was 0.723 (95% percentile interval 0.626–0.882). The differences between the empirical and bootstrap proportions, particularly for the Average Engagement and Attitude–Practice Constrained Profiles, indicated instability in estimated profile sizes.

**Figure 5:**
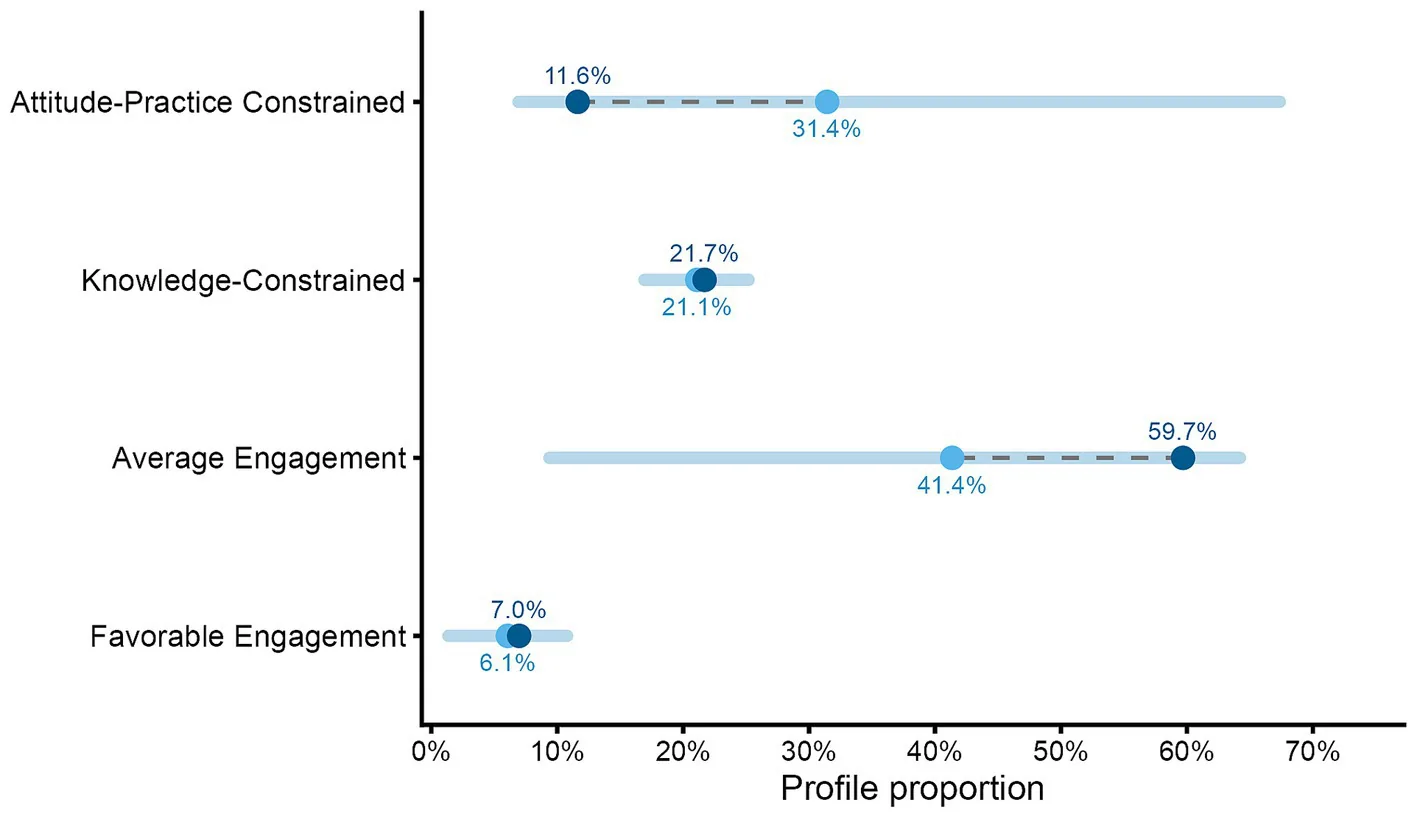
Bootstrap profile reproducibility. Comparison of empirical profile proportions with bootstrap estimates from 495 complete four-profile resamples. Dark-blue points represent empirical proportions, light-blue points represent mean bootstrap proportions, pale horizontal lines represent 95% percentile intervals, and dashed connectors indicate differences between the empirical and bootstrap means. Bootstrap labels were matched to the original profiles by selecting the permutation that minimized the total Euclidean distance across the three standardized KAP indicator means.

Class-specific bootstrap parameter distributions supported the high-Attitude and high-Practice pattern of Favorable Engagement and the low-Knowledge pattern of Knowledge-Constrained. In contrast, the wider distributions for Average Engagement and attenuation of the negative Attitude and Practice scores in the Attitude–Practice Constrained Profile indicated uncertainty in the magnitude and boundaries of these profiles. Complete parameter distributions are reported in Supplementary Table 7.

The Favorable Engagement pattern was reproduced in 98.8% of complete resamples, and the low-Knowledge pattern of the Knowledge-Constrained Profile was reproduced in 95.4%. The combined low-Attitude and low-Practice pattern of the Attitude–Practice Constrained Profile was reproduced in 62.6%, while the full distinction between the two constrained profiles was reproduced in 62.8%. Thus, some defining patterns were reproducible, whereas the separation and size estimates of other profiles remained unstable. Additional bootstrap results are provided in Supplementary Table 8.

When Knowledge was represented only by the reverse-coded awareness item, the overall evidence favored three rather than four profiles, indicating that the selected number and boundaries of profiles were sensitive to the operational definition of Knowledge (Supplementary Table 9).

## 4 Discussion

This study identified four exploratory exercise self-management profiles: Favorable Engagement, Average Engagement, Knowledge-Constrained, and Attitude–Practice Constrained. The main contribution was the distinction between limited knowledge-related access or awareness and combined attitude and practice constraints. BMI showed the clearest adjusted external association, whereas glycemic indicators and complications did not differ robustly. The chronic-disease association was sensitive to classification uncertainty.

The Knowledge-Constrained Profile had markedly low Knowledge-related scores but near-average Attitude and only mildly reduced Practice, suggesting a potential information or education-access gap. The Attitude–Practice Constrained Profile combined the lowest Attitude and Practice despite near-average Knowledge-related scores, suggesting that information alone may be insufficient. Because the latter pattern and the separation between constrained profiles were reproduced in only about 63% of bootstrap samples, these interpretations remain hypothesis-generating. These patterns may generate different intervention hypotheses, including improved access to exercise-management information for knowledge-related constraints and combined motivational and behavioral support for attitude–practice constraints (35–37). However, these implications require prospective testing because the constrained profiles showed limited bootstrap reproducibility.

BMI differed across profiles in adjusted models, but only the Average Engagement contrast persisted after classification uncertainty was incorporated. BMI reflects longer-term energy balance, yet diet, treatment, disease duration, and physical limitations were not fully measured. Direction and mechanism therefore cannot be established. Exercise may be related to adiposity through energy expenditure, skeletal-muscle glucose uptake, and metabolic or inflammatory pathways (38–40). However, these mechanisms were not measured in the present study, and higher BMI may itself reduce exercise capacity.

WHR, FBG, and HbA1c did not show significant adjusted profile differences. One possible explanation is that BMI reflects longer-term energy balance, whereas glycemic measures are more responsive to recent medication changes, dietary intake, treatment adherence, and measurement timing (41). This explanation remains speculative because these temporal factors were not directly measured. The null glycemic findings do not establish that exercise behavior is unrelated to glycemic control. They indicate only that the identified profiles did not distinguish concurrent glycemic status after adjustment and consideration of classification uncertainty.

Diabetes-related complications showed no robust profile association. The adjusted hard-assignment model suggested a difference in other chronic disease, but this finding did not persist in pseudo-class analyses and may reflect classification uncertainty or residual confounding. The profiles should not be interpreted as clinical risk strata.

Model selection remained a trade-off. The four-profile solution had the lowest AIC and provided additional behavioral differentiation, whereas the three-profile solution had the lowest BIC and SABIC and higher entropy. Four profiles were retained for exploratory resolution, not because all criteria favored them.

Bootstrap analyses reproduced the Favorable Engagement and Knowledge-Constrained patterns well, but profile-size estimates for Average Engagement and Attitude–Practice Constrained were variable. The awareness-only sensitivity analysis favored three profiles. Thus, the number and boundaries of profiles depended partly on measurement specification and sampling variation.

Several limitations should be considered. The cross-sectional design prevents causal or temporal inference, and some indicators were based on self-report. Residual confounding by disease severity, physical limitations, diabetes distress, depression, health literacy, healthcare access, or recruitment center remains possible. Participant-level center identifiers were unavailable, preventing adjustment for center effects or clustering. Counts of individuals screened, excluded, or declining participation and center-specific enrollment numbers were also unavailable. Consequently, a complete participant-flow diagram could not be reconstructed, limiting the assessment of potential selection bias. The Knowledge-related composite included educational exposure rather than a direct knowledge test, and the alternative Knowledge definition favored a different number of profiles.

The Attitude CFA showed mixed model fit and one inadmissible standardized loading. Classification quality was moderate, the Attitude–Practice Constrained Profile had the lowest APP, and five bootstrap samples yielded incomplete four-profile assignments. The analytic data also lacked sufficient information on the data sources, diagnostic confirmation procedures, and ascertainment time windows for recorded diabetes-related complications and other chronic disease; therefore, some uncertainty in outcome classification cannot be excluded. Sparse subtype events and recruitment from two regions further limit statistical precision and generalizability.

## 5 Conclusion

This multicenter cross-sectional study identified exploratory Knowledge-Constrained and Attitude–Practice Constrained exercise self-management patterns among adults with T2DM. BMI showed the clearest adjusted association, while glycemic and complication outcomes did not differ robustly. The profiles may inform hypotheses for tailored support but require prospective validation before clinical use.

## Data Availability

The datasets generated and/or analyzed during the current study are not publicly available due patient privacy protections and institutional restrictions but are available from the corresponding author on reasonable request. Requests to access the datasets should be directed to doctorxue001@foxmail.com.
