# Supplementary material for: Exercise self-management behavioral phenotypes in type 2 diabetes: a multicenter cross-sectional study
Source: Front Public Health. 2026 Sep 22;14:1892333. doi: 10.3389/fpubh.2026.1892333 (PMC13638545; doi:10.3389/fpubh.2026.1892333)
Supplement: Supplementary file 1 [file Table_1.DOCX]

**Supplementary Material 1. Survey Instruments**

**Section 1. General Information Questionnaire**

1. Age (years): ____
2. Sex: ☐ Male ☐ Female
3. Height (cm): ____	Weight (kg): ____	BMI: ____
4. Waist circumference (cm): ____	Hip circumference (cm): ____	WHR: ____
5. Education level: ☐Primary school or below	☐Junior high school	☐Senior high school / Technical secondary school	☐University / College	☐Master’s degree or above
6. Occupation type: ☐Farmer	☐Worker	☐Office staff / professional	☐Self-employed	☐Retired
   ☐Unemployed	☐Other
7. Working status: ☐Full-time	☐Part-time	☐Unemployed	☐Retired
8. Marital status: ☐Unmarried	☐Married	☐Divorced	☐Widowed
9. Ethnicity: ☐Han ethnicity ☐Hui ethnicity	☐Manchu ethnicity	☐Others
10. Religion: ☐None ☐Buddhism ☐Christianity ☐Islam ☐Other
11. Medical payment method: ☐Urban employee insurance	☐Urban resident insurance	☐New rural cooperative insurance ☐Commercial insurance ☐Out-of-pocket
12. Long-term residence: ☐Urban ☐Rural
13. Average monthly family income per capita: ☐ <2000 RMB	☐2000–4999 RMB	☐5000–7999 RMB	☐ ≥8000 RMB
14. Availability of community exercise support services: ☐ Yes ☐ No
    Sources of health support (multiple choice): ☐Family ☐Friends ☐Community ☐Health professionals ☐None ☐Other
15. Awareness of diabetes exercise management: ☐ Very well understood ☐ Relatively well understood ☐ Generally understood ☐ Not very well understood ☐ Completely unknown
16. Receipt of diabetes exercise health education: ☐ Yes ☐ No
17. Personality traits: ☐Introverted ☐Moderate ☐ Extroverted
18. Preference for physical activity: ☐Dislike strongly ☐Dislike ☐Neutral ☐Like ☐Like very much

## Section 2. Clinical and Disease-related Information

1.Duration of diabetes (years): ☐<1 year ☐1-5 years ☐ 5-10 years ☐>10 years

2. Number of hospitalizations: ☐ None ☐ 1–5 times ☐ 6–10 times ☐ >10 times
3. Family history of diabetes: ☐ Yes ☐ No
4. Smoking status: ☐Never ☐Former ☐Current
5. Alcohol consumption: ☐Never ☐Occasional ☐Regular ☐Everyday
6. History of hypoglycemia: ☐ Yes ☐ No (Frequency in last 2 weeks: _____)
7. Diabetic complications (multiple choice): ☐Yes ☐No
   ☐Ketosis acidosis ☐Diabetic nephropathy ☐Cardiovascular and cerebrovascular diseases ☐Hyperosmolar hyperglycemic state ☐Retinopathy ☐Lower extremity vascular disease ☐Diabetic foot ☐Lactic acidosis ☐Peripheral neuropathy ☐Gastroparesis ☐Other: _____Other chronic diseases (multiple choice): ☐ Yes ☐ No
   ☐Hypertension ☐Tumor ☐COPD ☐Chronic gastritis ☐Arthritis ☐Coronary heart disease ☐Heart failure ☐Cerebral infarction ☐TIA ☐Cerebral hemorrhage ☐Other: _____
8. Latest fasting plasma glucose (mmol/L): _____
9. Latest HbA1c (%): _____
10. Current treatment regimens (multiple choice):
    ☐Diet control ☐Exercise therapy ☐Oral hypoglycemic agents ☐Insulin therapy ☐Insulin + oral agents
    ☐Other

## Section 3. Exercise Belief Questionnaire (HBM-based)

## *Items are rated on a 5-point Likert scale: 1=strongly disagree, 2=disagree, 3=neutral, 4=agree, 5=strongly agree.*

| **Number** | **Question** | **Strongly disagree** | **disagree** | **neutral** | **agree** | **strongly agree** |
| --- | --- | --- | --- | --- | --- | --- |
| 1 | Without exercise self-management, my blood glucose control will deteriorate. |  |  |  |  |  |
| 2 | Without exercise self-management, I may develop acute or chronic complications of diabetes. |  |  |  |  |  |
| 3 | Without exercise self-management, I am more likely to develop other chronic diseases such as stroke or coronary heart disease. |  |  |  |  |  |
| 4 | Poor diabetes control would seriously affect my health. |  |  |  |  |  |
| 5 | Declining health would affect my daily life and social activities. |  |  |  |  |  |
| 6 | Declining health would increase the burden on my family. |  |  |  |  |  |
| 7 | Exercise self-management helps maintain and improve my health. |  |  |  |  |  |
| 8 | Exercise improves my overall well-being. |  |  |  |  |  |
| 9 | Exercise alleviates diabetes-related discomfort. |  |  |  |  |  |
| 10 | Exercise helps prevent and control diabetes complications. |  |  |  |  |  |
| 11 | I lack suitable facilities/equipment for exercise. |  |  |  |  |  |
| 12 | I do not receive professional guidance for exercise. |  |  |  |  |  |
| 13 | Physical discomfort limits my exercise. |  |  |  |  |  |
| 14 | I am worried that exercise may cause hypoglycemia or injury. |  |  |  |  |  |
| 15 | I have no time to exercise. |  |  |  |  |  |
| 16 | Doctors encourage me to exercise. |  |  |  |  |  |
| 17 | My family and friends encourage me to exercise. |  |  |  |  |  |
| 18 | Media promotes the benefits of exercise. |  |  |  |  |  |
| 19 | I am confident that I can adhere to exercise self-management. |  |  |  |  |  |
| 20 | I can usually solve problems encountered during exercise. |  |  |  |  |  |
| 21 | Exercise self-management is easy for me to perform. |  |  |  |  |  |

## Section 4. Exercise Self-Management Behavior Scale

*Response options: Never / Sometimes / Often / Always*

| **Number** | **Question** | **Never** | **Sometimes** | **Often** | **Always** |
| --- | --- | --- | --- | --- | --- |
| 1 | Have you developed an individualized exercise plan with healthcare professionals? |  |  |  |  |
| 2 | Do you exercise even when you are busy? |  |  |  |  |
| 3 | Do you perform warm-up and cool-down exercises? |  |  |  |  |
| 4 | Do you measure blood glucose before exercise? |  |  |  |  |
| 5 | Do you check the safety of exercise equipment and environment? |  |  |  |  |
| 6 | Do you consider weather and temperature before exercise? |  |  |  |  |
| 7 | Do you wear appropriate clothing and footwear? |  |  |  |  |
| 8 | Do you carry sugar or snacks in case of hypoglycemia? |  |  |  |  |
| 9 | Do you monitor heart rate or step count during exercise? |  |  |  |  |
| 10 | Do you stop exercising when discomfort occurs? |  |  |  |  |
| 11 | Do you exercise about one hour after meals? |  |  |  |  |
| 12 | Do you exercise 3–5 days per week? |  |  |  |  |
| 13 | Do you exercise at least 30 minutes per day? |  |  |  |  |
| 14 | Do you mainly perform moderate-intensity activity? |  |  |  |  |
| 15 | Do you inspect your feet after exercise? |  |  |  |  |
| 16 | Do you seek medical help if foot problems occur? |  |  |  |  |
| 17 | Do your family and friends support your exercise? |  |  |  |  |
| 18 | Do you regularly evaluate the effectiveness of your exercise program? |  |  |  |  |

**Supplementary Material 2. Tables**

Measurement and scoring notes. The full exercise-belief questionnaire contains 21 items. The Attitude CFA used eight prespecified items: three perceived-benefit items, two perceived-barrier items, and three self-efficacy items. Awareness was originally coded 1 = very well understood to 5 = completely unknown and was reverse-coded before standardization. Education was coded 1 = no and 2 = yes. Higher component scores therefore indicated greater awareness or educational exposure.

**Supplementary Table 1. Model-Specification Checks for the Latent Profile Analysis**

| **Model** | **Profiles** | **AIC** | **BIC** | **SABIC** | **Entropy** | **LogLik** | **BLRT p** |
| --- | --- | --- | --- | --- | --- | --- | --- |
| 1 | 2 | 4316.77 | 4359.23 | 4327.49 | 0.824 | -2148.39 | 0.0099 |
| 1 | 3 | 4113.30 | 4172.74 | 4128.30 | 0.852 | -2042.65 | 0.0099 |
| **1** | **4** | **4109.58** | **4186.01** | **4128.88** | **0.714** | **-2036.79** | **0.0099** |
| 2 | 2 | 4173.73 | 4228.92 | 4187.66 | 0.912 | -2073.86 | 0.0099 |
| 2 | 3 | 4104.56 | 4189.48 | 4126.00 | 0.851 | -2032.28 | 0.0099 |
| 2 | 4 | 4075.24 | 4189.88 | 4104.18 | 0.718 | -2010.62 | 0.0099 |
| 3 | 2 | 4109.30 | 4164.50 | 4123.23 | 0.937 | -2041.65 | 0.0099 |
| 3 | 3 | 4099.68 | 4171.87 | 4117.91 | 0.858 | -2032.84 | 0.0099 |
| 3 | 4 | 4099.30 | 4188.47 | 4121.81 | 0.739 | -2028.65 | 0.0495 |

Note. Model 1 assumes equal within-profile variances and zero within-profile covariances; Model 2 allows within-profile variances to vary and fixes covariances to zero; Model 3 assumes equal variances and equal covariances across profiles. Model 1 was the primary parameterization; Models 2 and 3 were supplementary model-specification checks. The selected four-profile Model 1 solution is highlighted. BLRT compares the k-profile solution with the k−1-profile solution. BLRT p values were based on 100 bootstrap draws and may show minor Monte Carlo variation. AIC, Akaike information criterion; BIC, Bayesian information criterion; SABIC, sample-size-adjusted Bayesian information criterion; BLRT, bootstrap likelihood-ratio test.

**Supplementary Table 2. Continuous Clinical Outcomes by Assigned Profile**

| **Outcome** | **Favorable Engagement** | **Average Engagement** | **Knowledge-Constrained** | **Attitude–Practice Constrained** |
| --- | --- | --- | --- | --- |
| BMI | 22.34 ± 2.91 | 25.03 ± 4.14 | 24.08 ± 3.99 | 25.15 ± 5.03 |
| WHR | 0.86 ± 0.07 | 0.92 ± 0.19 | 0.89 ± 0.11 | 0.92 ± 0.08 |
| FBG | 7.18 ± 0.99 | 7.27 ± 2.00 | 7.30 ± 1.63 | 8.02 ± 2.61 |
| HbA1c | 7.05 ± 0.82 | 7.22 ± 1.78 | 7.16 ± 1.96 | 7.36 ± 1.70 |

No outcome data were missing for the six clinical outcomes.

**Supplementary Table 3. Binary Clinical Outcomes by Assigned Profile**

| **Outcome** | **Favorable Engagement** | **Average Engagement** | **Knowledge-Constrained** | **Attitude–Practice Constrained** |
| --- | --- | --- | --- | --- |
| Complication | 2/36 (5.6%; 1.5–18.1) | 76/308 (24.7%; 20.2–29.8) | 33/112 (29.5%; 21.8–38.5) | 19/60 (31.7%; 21.3–44.2) |
| Other chronic disease | 11/36 (30.6%; 18.0–46.9) | 153/308 (49.7%; 44.1–55.2) | 64/112 (57.1%; 47.9–65.9) | 40/60 (66.7%; 54.1–77.3) |

**Supplementary Table 4A. Adjusted Continuous-Outcome Models**

| **Outcome** | **Contrast vs Favorable** | **Measure** | **Estimate** | **95% CI** | **p** | **Overall p** |
| --- | --- | --- | --- | --- | --- | --- |
| BMI | Average Engagement | beta | 2.70 | 1.18 to 4.23 | 0.001 | 0.002 |
| BMI | Knowledge-Constrained | beta | 1.79 | 0.12 to 3.45 | 0.036 | 0.002 |
| BMI | Attitude–Practice Constrained | beta | 2.81 | 1.00 to 4.63 | 0.003 | 0.002 |
| WHR | Average Engagement | beta | 0.06 | 0.00 to 0.12 | 0.037 | 0.108 |
| WHR | Knowledge-Constrained | beta | 0.04 | -0.03 to 0.10 | 0.260 | 0.108 |
| WHR | Attitude–Practice Constrained | beta | 0.07 | -0.00 to 0.13 | 0.064 | 0.108 |
| FBG | Average Engagement | beta | 0.01 | -0.69 to 0.72 | 0.971 | 0.073 |
| FBG | Knowledge-Constrained | beta | 0.03 | -0.74 to 0.80 | 0.942 | 0.073 |
| FBG | Attitude–Practice Constrained | beta | 0.73 | -0.11 to 1.57 | 0.090 | 0.073 |
| HbA1c | Average Engagement | beta | 0.13 | -0.51 to 0.77 | 0.689 | 0.611 |
| HbA1c | Knowledge-Constrained | beta | 0.02 | -0.67 to 0.71 | 0.956 | 0.611 |
| HbA1c | Attitude–Practice Constrained | beta | 0.38 | -0.37 to 1.14 | 0.323 | 0.611 |

**Supplementary Table 4B. Adjusted Binary-Outcome Models**

| **Outcome** | **Contrast vs Favorable** | **Measure** | **Estimate** | **95% CI** | **p** | **Overall p** |
| --- | --- | --- | --- | --- | --- | --- |
| complication | Average Engagement | OR | 2.15 | 0.46 to 10.01 | 0.327 | 0.437 |
| complication | Knowledge-Constrained | OR | 2.82 | 0.58 to 13.64 | 0.198 | 0.437 |
| complication | Attitude–Practice Constrained | OR | 2.81 | 0.55 to 14.40 | 0.214 | 0.437 |
| other chronic disease | Average Engagement | OR | 1.87 | 0.84 to 4.16 | 0.126 | 0.040 |
| other chronic disease | Knowledge-Constrained | OR | 2.28 | 0.96 to 5.44 | 0.062 | 0.040 |
| other chronic disease | Attitude–Practice Constrained | OR | 3.62 | 1.39 to 9.47 | 0.009 | 0.040 |

**Supplementary Table 5. Posterior-Probability Pseudo-Class Models**

| **Outcome** | **Contrast vs Favorable** | **Measure** | **Estimate** | **95% CI** | **p** |
| --- | --- | --- | --- | --- | --- |
| Any other chronic disease | Average Engagement | OR | 1.98 | 0.80 to 4.94 | 0.141 |
| Any complication | Average Engagement | OR | 1.64 | 0.31 to 8.84 | 0.562 |
| BMI | Average Engagement | beta | 1.96 | 0.25 to 3.67 | 0.025 |
| FBG | Average Engagement | beta | 0.00 | -0.78 to 0.78 | 0.997 |
| HbA1c | Average Engagement | beta | 0.00 | -0.69 to 0.70 | 0.992 |
| WHR | Average Engagement | beta | 0.02 | -0.08 to 0.11 | 0.758 |
| Any other chronic disease | Knowledge-Constrained | OR | 2.25 | 0.89 to 5.70 | 0.087 |
| Any complication | Knowledge-Constrained | OR | 2.20 | 0.41 to 11.69 | 0.356 |
| BMI | Knowledge-Constrained | beta | 1.06 | -0.71 to 2.83 | 0.239 |
| FBG | Knowledge-Constrained | beta | 0.03 | -0.79 to 0.85 | 0.945 |
| HbA1c | Knowledge-Constrained | beta | -0.11 | -0.84 to 0.61 | 0.755 |
| WHR | Knowledge-Constrained | beta | -0.01 | -0.10 to 0.08 | 0.854 |
| Any other chronic disease | Attitude–Practice Constrained | OR | 2.31 | 0.81 to 6.59 | 0.119 |
| Any complication | Attitude–Practice Constrained | OR | 1.78 | 0.32 to 9.85 | 0.507 |
| BMI | Attitude–Practice Constrained | beta | 1.94 | -0.07 to 3.96 | 0.059 |
| FBG | Attitude–Practice Constrained | beta | 0.23 | -0.68 to 1.14 | 0.616 |
| HbA1c | Attitude–Practice Constrained | beta | 0.05 | -0.77 to 0.86 | 0.910 |
| WHR | Attitude–Practice Constrained | beta | 0.01 | -0.09 to 0.11 | 0.829 |

**Supplementary Table 6. Complication-Subtype Analyses**

| **Subtype** | **Favorable Engagement** | **Average Engagement** | **Knowledge-Constrained** | **Attitude–Practice Constrained** | **Fisher p** | **FDR p** |
| --- | --- | --- | --- | --- | --- | --- |
| Ketosis Acidosis | 0/36 (0.0%) | 1/308 (0.3%) | 0/112 (0.0%) | 0/60 (0.0%) | 1.000 | 1.000 |
| Nephropathy | 1/36 (2.8%) | 25/308 (8.1%) | 6/112 (5.4%) | 8/60 (13.3%) | 0.224 | 0.624 |
| Cardiovascular Cerebrovascular | 0/36 (0.0%) | 17/308 (5.5%) | 11/112 (9.8%) | 5/60 (8.3%) | 0.134 | 0.624 |
| Hyperosmolar State | 0/36 (0.0%) | 1/308 (0.3%) | 1/112 (0.9%) | 0/60 (0.0%) | 0.644 | 0.716 |
| Retinopathy | 0/36 (0.0%) | 26/308 (8.4%) | 7/112 (6.2%) | 5/60 (8.3%) | 0.292 | 0.624 |
| Lower Extremity Vascular | 0/36 (0.0%) | 11/308 (3.6%) | 5/112 (4.5%) | 0/60 (0.0%) | 0.328 | 0.624 |
| Diabetic Foot | 0/36 (0.0%) | 6/308 (1.9%) | 4/112 (3.6%) | 0/60 (0.0%) | 0.437 | 0.624 |
| Lactic Acidosis | 0/36 (0.0%) | 1/308 (0.3%) | 1/112 (0.9%) | 0/60 (0.0%) | 0.644 | 0.716 |
| Peripheral Neuropathy | 1/36 (2.8%) | 29/308 (9.4%) | 11/112 (9.8%) | 8/60 (13.3%) | 0.399 | 0.624 |
| Gastroparesis | 0/36 (0.0%) | 0/308 (0.0%) | 1/112 (0.9%) | 0/60 (0.0%) | 0.403 | 0.624 |

**Supplementary Table 7. Empirical and Bootstrap Distributions of Standardized KAP Indicator Means Across the Four Profiles**

| **Profile** | **Indicator** | **Empirical mean** | **Bootstrap mean** | **95% percentile interval** |
| --- | --- | --- | --- | --- |
| **Favorable Engagement** | Knowledge | 0.293 | 0.021 | −1.734 - 0.606 |
|  | Attitude | 1.825 | 1.923 | 1.454 - 2.547 |
|  | Practice | 1.785 | 1.885 | 1.274 - 2.385 |
| **Average Engagement** | Knowledge | 0.555 | 0.557 | 0.419 - 0.765 |
|  | Attitude | 0.033 | 0.444 | −0.142 - 1.560 |
|  | Practice | 0.088 | 0.347 | −0.042 - 1.398 |
| **Knowledge-Constrained** | Knowledge | −1.649 | −1.664 | −1.846 - −1.486 |
|  | Attitude | 0.002 | −0.003 | −0.175 - 0.164 |
|  | Practice | −0.358 | −0.393 | −0.609 - −0.207 |
| **Attitude–Practice Constrained** | Knowledge | 0.053 | 0.250 | −0.315 - 0.514 |
|  | Attitude | −1.268 | −0.860 | −1.372 - −0.227 |
|  | Practice | −0.855 | −0.502 | −1.035 - −0.083 |

Values are standardized means. Bootstrap means and 95% percentile intervals were calculated from 495 complete four-profile bootstrap resamples after permutation-based label alignment. KAP, knowledge, attitude, and practice.

**Supplementary Table 8. Bootstrap Reproducibility**

| **Profile** | **Empirical** | **Bootstrap mean** | **95% percentile interval** |
| --- | --- | --- | --- |
| Favorable Engagement | 7.0% | 6.1% | 1.4%–10.8% |
| Average Engagement | 59.7% | 41.4% | 9.4%–64.2% |
| Knowledge-Constrained | 21.7% | 21.1% | 16.9%–25.2% |
| Attitude–Practice Constrained | 11.6% | 31.4% | 6.9%–67.4% |

Pattern reproduction: Favorable Engagement 98.8%; Knowledge-Constrained 95.4%; combined lowest Attitude and Practice 62.6%; full distinction between constrained profiles 62.8%. Of 500 resamples, 495 were complete. Mean entropy was 0.723 (95% percentile interval 0.626–0.882).

**Supplementary Table 9. Awareness-Only Sensitivity Analysis**

| **Profiles** | **AIC** | **BIC** | **SABIC** | **Entropy** | **BLRT p** | **Class sizes** | **APP** |
| --- | --- | --- | --- | --- | --- | --- | --- |
| 3 | 4307.32 | 4366.76 | 4322.33 | 0.631 | 0.0099 | 310/165/41 | 0.839/0.768/0.854 |
| 4 | 4303.74 | 4380.17 | 4323.04 | 0.530 | 0.0891 | 214/143/122/37 | 0.706/0.698/0.753/0.834 |

Note. Knowledge was represented only by the reverse-coded five-level awareness item; the education-exposure item was excluded. APP, average posterior probability.

**Supplementary material 3: Standardized Fac -r Loadings and Fac -r Correlations for the Attitude CFA**

(1) **scaled χ² = 65.218**
(2) **df = 17**
(3) **p < 0.001**
(4) **robust CFI = 0.977**
(5) **robust TLI = 0.962**
(6) **robust RMSEA = 0.078**
(7) **robust RMSEA 95% CI = 0.059–0.099**
(8) **SRMR = 0.029**
